# Severe *emm89* Group A *Streptococcal* Disease Characterized by Toxic Shock and Endocarditis

**DOI:** 10.1155/2019/6568732

**Published:** 2019-01-21

**Authors:** Morouge Alramadhan, Gloria P. Heresi, Anthony R. Flores

**Affiliations:** ^1^Division of Infectious Diseases, Department of Pediatrics, McGovern Medical School, University of Texas Health Sciences Center at Houston, Houston, USA; ^2^Center for Antimicrobial Resistance and Microbial Genomics, McGovern Medical School, University of Texas Health Sciences Center at Houston, Houston, USA

## Abstract

Invasive group A *Streptococcus* infections are associated with diverse presentations. We report a severe, rare case of GAS infection with dissemination including endocarditis and STSS. While whole genome sequencing of blood and pharyngeal isolates did not reveal any unique features attributable to the severe presentation, our approach serves as a template for investigation of severe manifestations of common infections.

## 1. Introduction

Invasive group A *Streptococcus* (iGAS) infections are associated with high morbidity and mortality. Approximately 11,000 to 13,000 cases of iGAS infections occur every year in the United States with varying case fatality rates (CFRs) depending on the associated syndrome [1]. Clinical syndromes of iGAS infections with the highest CFR include streptococcal toxic-shock syndrome (STSS; ∼40% CFR) and necrotizing fasciitis (NF; ∼30% CFR) compared to localized skin and soft tissue infections (SSTIs) or abscesses with much lower mortality (<5% CFR). Thus, prompt recognition and aggressive medical, and if necessary surgical, management are paramount in mitigating complications and death.

Substantial research efforts have been devoted in defining GAS strain-disease associations. GAS are categorized based on >200 *emm* (gene encoding the antiphagocytic M protein) types. In most countries, *emm* types 1, 3, 12, and 28 have traditionally been associated with invasive GAS disease. Recently, *emm89* GAS emergence was found to be associated with simultaneous loss of capsule biosynthesis genes and acquisition of *emm1*-like *nga/slo* alleles resulting in enhanced cytotoxic activity [2]. The “new” *emm89* epidemic clone now comprises the majority of *emm89*-associated disease and is second to only *emm1* in frequency of iGAS [1].

Here, we describe a severe, rare presentation of patients with STSS and disseminated infection including right-sided endocarditis. Using bacterial whole genome sequencing (WGS), we molecularly characterized the pharyngeal and invasive GAS case isolates from the same patient.

## 2. Case Report

A 6-year-old boy, with a history of recurrent throat infections presented to Children's Memorial Hermann Hospital (CMHH) following 5 days of fever, sore throat, nasal congestion, and cough. Prior to presentation to CMHH, on day 1 of illness, he was diagnosed with influenza infection (clinical diagnosis) by his primary care physician (PCP) and prescribed oseltamivir which was discontinued on day 3 due to nausea and vomiting. Subsequently, on the 5^th^ day of illness, he started to have abdominal and joint pain (left knee, right ankle, and right elbow). He was noted to be lethargic and had decreased oral intake with dark urine and, thus, was brought to CMHH emergency center (EC). In the CMHH EC, he was febrile (T 39.4°C), hypotensive (blood pressure 78/47 mm Hg), tachypneic (respiratory rate 33 per minute), and tachycardic (heart rate 160 per minute). He was admitted to a pediatric intensive care unit and was started on intravenous cefepime and vancomycin empirically. He lives with his mother and 3 siblings (aged 2, 9, and 10 years), all of whom were healthy with no current or prior symptoms.

Physical examination revealed an acutely ill but responsive boy with crusted lip lesions, cervical lymphadenopathy, nasal congestion, nonpurulent pharyngeal erythema, systolic murmur, and hepatosplenomegaly and no sign of arthritis. Initial lab studies showed a normal white blood cell (WBC) count (11,600/mm^3^) with an unremarkable differential, thrombocytopenia (36,000/mm^3^), anemia (hemoglobin 8.9 g/dL), hypoalbuminemia (1.9 g/dL), proteinuria (100 mg/dL), sterile pyuria (WBC 21/high powered field), and markedly elevated inflammatory markers (C-reactive protein 182 mg/L; erythrocyte sedimentation rate >100 mm/hr). Blood cultures drawn on admission along with pharyngeal culture were positive for GAS within 24 hours.

**Figure 1:**
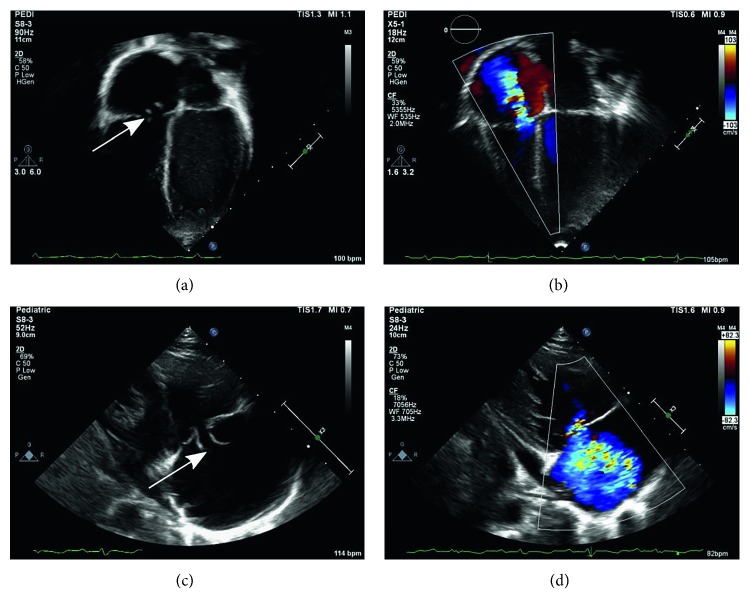
Echocardiogram showing echocardiographic clips: (a) two-dimensional apical 4-chamber view showing tricuspid valve leaflet perforation (arrow) with possible chordal disruption, (b) color Doppler image of apical 4-chamber view showing tricuspid regurgitation (blue jet), (c) two-dimensional parasternal long-axis view showing tricuspid valve leaflet perforation (arrow), and (d) color Doppler image of parasternal long-axis view showing tricuspid regurgitation (blue jet).

An echocardiogram was obtained due to the severity of presentation, presence of a murmur, and concern for infective endocarditis (IE). The transthoracic echocardiogram revealed echogenic tissues on the septal leaflet of the tricuspid valve (both on septal and anterior leaflets), tricuspid valve leaflet perforation with possible chordal disruption indicative of IE (Figure 1). Chest radiographs were normal throughout the admission. Magnetic resonance imaging (MRI) of the body was obtained to evaluate for foci of dissemination and revealed bilateral nephromegaly with bilateral wedge-shaped areas of abnormal signal (possible pyelonephritis and/or thromboembolic disease), osteomyelitis involving the right inferior pubic rami and right obturator externus muscle abscess, periportal edema with hepatosplenomegaly, and bilateral pleural effusions.

Meeting the diagnostic criteria for STSS (hypotension with coagulopathy (thrombocytopenia), pleural/peritoneal effusions with hypoalbuminemia, myositis, and isolation of GAS), his antimicrobial therapy was changed to intravenous penicillin G and clindamycin. Subsequent blood cultures were negative. He did not receive intravenous immunoglobulin. Consultations with cardiology and orthopedic surgery were obtained, but no interventions were needed during hospitalization. He progressively improved and was treated with clindamycin for 1 week and penicillin G IV for 6 weeks to treat GAS IE with close cardiology follow-up for possible tricuspid valve repair.

**Figure 2:**
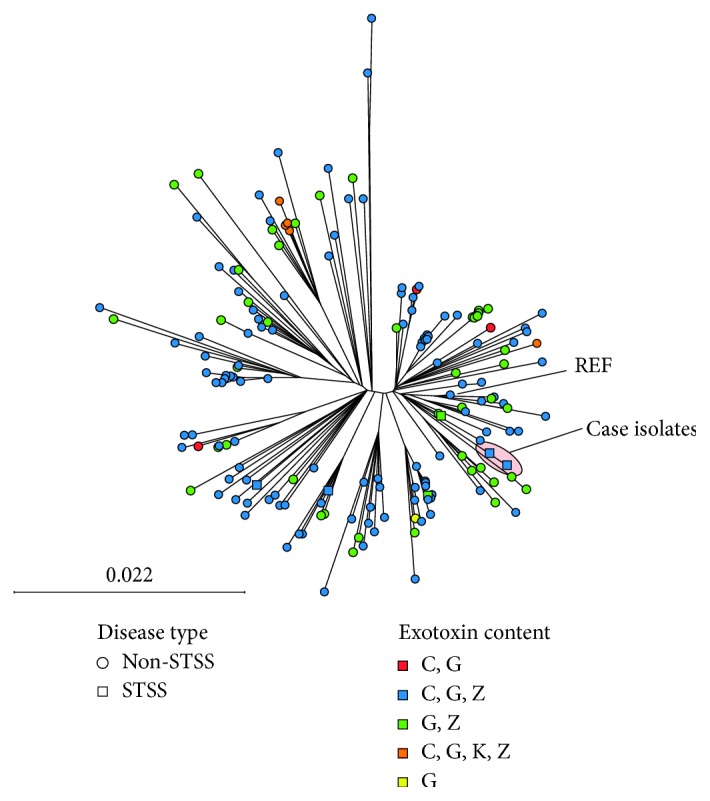
Neighbor-joining phylogenetic tree based on 2,464 unique, core genome biallelic single nucleotide (SNP) differences relative to the reference, MGAS27061, between case (circled) and CDC-sequenced *emm89* iGAS strains from 2015 [4]. GAS isolates from cases of toxic shock are depicted by boxes including 2 from the 2015 CDC-sequenced strains and 2 previously described isolates from Houston, TX.

This is particularly unusual presentation in an otherwise healthy child. GAS invasive disease may be associated with specific GAS clones harboring genetic elements or mutations that predispose to severe disease. Given the severity of diseases (STSS) and unusual multifocal presentation with endocarditis, we performed bacterial WGS, as we have previously described [3], to define the bacterial strain characteristics associated with the pharyngeal and blood strains. The pharyngeal and blood GAS isolates from the patient were identified as *emm89* GAS and were identical at the nucleotide level apart from a pentanucleotide repeat (10 bp deletion) in the gene encoding streptococcal collagen-like protein B (SclB) of the pharyngeal isolate compared to the blood. However, the deletion is not predicted to alter SclB expression as both isolates maintain a frame-shift mutation in *SclB* and thus likely do not express the protein. We did not identify mutations in virulence gene regulators such as *covRS* known to lead to hypervirulence. Comparing the case isolates to recent GAS WGS performed by the Centers for Disease Control and Prevention (CDC) [4] revealed a similar clonal relationship to nationally circulating *emm89* iGAS (Figure 2). The case of pharyngeal and blood isolates did not have a unique *streptococcal pyrogenic exotoxin* (*spe*) gene content compared to non-STSS iGAS in the CDC collection and was similar to *emm89* STSS strains in Houston, TX (Figure 2).

## 3. Discussion

Here, we describe a rare case of STSS and dissemination including right-sided endocarditis due to *emm89* GAS. We used bacterial WGS analysis to show that the case *emm89* strains (pharyngeal and blood) are essentially identical and do not contain unusual gene content or polymorphisms that we can readily attribute to this unusual presentation of iGAS. Furthermore, it does not appear that our *emm89* GAS case strains are unique in which they are similar to circulating *emm89* iGAS recently sequenced by the CDC. For example, the case strains contain similar exotoxin gene content compared to national CDC surveillance. Studies suggest individuals with specific HLA genotypes may be more susceptible to severe presentations of iGAS disease [5]. However, most likely, our case represents one extreme of a very broad distribution of iGAS presentation. Further research is needed to elucidate the pathogen and host factors that contribute to severe iGAS clinical presentations.

Our case teaches a few important points. First, infective endocarditis due to GAS infections is incredibly unusual. Including a report of 2 cases in 2014, a total of 15 pediatric cases of infective endocarditis due to GAS infections have been reported since 1942 [6]. No previous case reports included GAS strain characteristics. Interestingly, only one previous published case report involved the right-sided tricuspid valve and the others all involved the left-sided heart valves (mitral and aortic). Thus, while we can only speculate, endocarditis in our patient is likely secondary following dissemination from primary pharyngeal or other (e.g., bone) infections.

Second, our case illustrates the utility of bacterial WGS in strain characterization. Advances in sequencing technologies and bioinformatic analyses coupled with rapidly decreasing costs mean that infectious disease practitioners will begin to utilize and be confronted with WGS data more frequently for outbreak and single bacterial strain investigations. The utility of publicly available iGAS WGS recently made available by the CDC's Active Bacterial Core Surveillance cannot be overstated. While bacterial WGS did not identify unique strain characteristics for the unusual presentation and severity of infection, we were able to show that the case strains were similar to circulating *emm89* GAS strains in the United States but distinct from other known *emm89* STSS strains. Thus, it is unlikely that our case represents an outbreak or emergence of a hypervirulent clone.

Finally, increasingly, data suggest antibiotic prophylaxis for close contacts of iGAS cases. Some suggest antibiotic prophylaxis for those exposed to an index patient 24 h/week [7]. However, current guidelines do not recommend routine antibiotic prophylaxis, and this is likely to remain at the discretion of the individual practitioner [8]. All close contacts of our case were evaluated for GAS infections by throat culture and found to be negative except a 10-year-old asymptomatic brother who was treated with cefdinir for 10 days.

In summary, our case represents a very rare and severe presentation of iGAS disease. The use of bacterial WGS rapidly determined strain characteristics and confirmed similarity to circulating *emm89* iGAS in the United States, alleviating the concern of a hypervirulent clone emergence.
